# Barriers and enablers to the implementation of one health strategies in developing countries: a systematic review

**DOI:** 10.3389/fpubh.2023.1252428

**Published:** 2023-11-23

**Authors:** Daniele Sandra Yopa, Douglas Mbang Massom, Gbètogo Maxime Kiki, Ramde Wendkoaghenda Sophie, Sylvie Fasine, Oumou Thiam, Lassane Zinaba, Patrice Ngangue

**Affiliations:** ^1^Faculty of Medicine and Biomedical Sciences, University of Yaounde 1, Yaoundé, Cameroon; ^2^Faculty of Medicine and Pharmaceutical Sciences, University of Douala, Douala, Cameroon; ^3^Rehabilitation Department, Université Laval, Québec, QC, Canada; ^4^Center for Interdisciplinary Research in Rehabilitation and Social Integration, Québec, QC, Canada; ^5^Ministry of Health and Public Hygiene, Ouagadougou, Burkina Faso; ^6^National Institute of Biomedical Research, Ministry of Public Health, Kinshasa, Democratic Republic of Congo; ^7^Ministry of Health and Public Hygiene, Conakry, Guinea; ^8^Institute for Interdisciplinary Training and Research in Health Sciences and Education, Ouagadougou, Burkina Faso; ^9^Faculty of Nursing Sciences, Université Laval, Québec, QC, Canada

**Keywords:** barriers, enablers, one health, strategies, developing countries, multisectoral collaboration

## Abstract

**Introduction:**

One Health is a concept that establishes the link between humans, animals and the environment in a collaborative approach. Since One Health’s inception, several interventions have been developed in many regions and countries worldwide to tackle complex health problems, including epidemics and pandemics. In the developed world, many collaborative platforms have been created with an international strategy to address issues specific or not to their environment. Unfortunately, there is a lack of synthesis on the challenges and opportunities Low and Middle-Income Countries (LMICs) face.

**Methods:**

Following The Preferred Reporting Elements for PRISMA Systematic Reviews and Meta-Analyses (PRISMA), we conducted a systematic review. We applied a search strategy to electronic bibliographic databases (PubMed, Embase, Global Health, Web of Science and CINAHL). We assessed the included articles’ quality using the Mixed Methods Appraisal tool (MMAT).

**Results and discussion:**

A total of 424 articles were initially identified through the electronic database search. After removing duplicates (n = 68), 356 articles were screened for title and abstract, and 16 were retained for full-text screening. The identified barriers were the lack of political will, weak governance and lack of human, financial and logistics resources. Concerning the enablers, we listed the existence of a reference framework document for One Health activities, good coordination between the different sectors at the various levels, the importance of joint and multisectoral meetings that advocated the One Health approach and the Availability of funds and adequate resources coupled with the support of Technical and Financial partners.

**Conclusion:**

One Health strategy and interventions must be implemented widely to address the rising burden of emerging infectious diseases, zoonotic diseases, and antimicrobial resistance. Addressing those challenges and reinforcing the enablers to promote managing global health challenges is necessary.

**Systematic Review Registration:**

https://www.crd.york.ac.uk/prospero/record_email.php, Unique Identifier: CRD42023393693.

## 1 Introduction

In recent years, the One Health approach has gained increasing attention as a means of addressing complex global health challenges. The said approach indicates that human, animal, and environmental health are inextricably related and interconnected, so protecting one’s health inevitably affects the other. One Health is a concept that first derived from Conservation Medicine through the Manhattan principles and was later involved in health experts’ discussions on Planetary Health and their commitment to preserving the link between the different elements of the ecosystem (1, 2). It is an approach that reinforces the Sustainable Development Goals (SDGs) ambitions and helps accelerate progress toward their achievement by promoting good health (3). Its main objective is to improve the health of humans, animals, and the environment (4). It is implemented as a collaborative approach between the different actors of society at various levels. It integrates several principles, including equity between sectors and disciplines, sociopolitical and multicultural parity, socio-ecological equilibrium, transdisciplinarity, and multisectoral collaboration (5, 6).

Since One Health’s inception, it has gained significant attention due to its integrated and holistic approaches, which fit well with the current disease trends involving humans, animals and the environment (7). Several interventions have been developed in many regions and countries worldwide to tackle complex health problems, including epidemics and pandemics. In the developed world, many collaborative platforms have been created with an international strategy to address issues specific or not to their environment (8). In addition, it has been determined that the COVID-19 pandemic governance could be improved by applying One Health strategies, such as surveillance and monitoring of the occurrence of infectious diseases in both humans and animals (9).

Effective implementation of One Health programs can only be well implemented and successful by embedding together political commitment, policy formulation, sustainable financing, program development, knowledge sharing, institutional collaboration, capacity enhancement, engagement of civil society, and active participation of the communities (10, 11). Interdisciplinary collaboration requires several actors in different domains (human, animal and environmental health) to work together. Other challenges, such as divergent interests, conflicting priorities, siloes, and lack of trust, have been reported as the main obstacles to the success of One Health programs (12–14). Although several actions have been implemented to reduce the global health burden in Low and Middle-Income Countries (LMICs), they still need to improve their healthcare systems, which remain among the weakest and most inefficient in the world (15, 16). So far, a minimal number of LMICs have been able to put in place concrete and effective programs that promote the implementation of strategies in the control of emerging and future health issues in the human, animal, and environmental sectors, therefore highlighting the need for them to prioritize One Health (17). The numerous public health disasters experienced and managed in LMICs, such as African countries, have their origins in animal populations and are linked with agroecological change. In addition, the maturing process of most medical and veterinary institutions represents an opportunity to better understand the inextricable link between the human, animal, and ecological health sectors and position itself as a promising One Health hub (18).

Many authors have analyzed the challenges the developing world faces in adopting a multisectoral approach in response to public health issues and controlling disease outbreaks. However, there needs to be a synthesis of these challenges and opportunities in LMICs. This systematic review aimed to identify barriers and enablers in implementing One Health strategies in LMICs. The findings of this review will help identify the root of the matter globally and inform us on the potential actions to initiate. Furthermore, addressing the barriers and building on the facilitators will provide a pathway to achieving the goal of One Health and ensure the health and well-being of human, animal and environmental health for current and future generations.

## 2 Materials and methods

This systematic review followed The Preferred Reporting Elements for PRISMA Systematic Reviews and Meta-Analyses (19, 20). This systematic review protocol was registered in the International Register of Prospective Systematic Reviews (PROSPERO), CRD42023393693, on February 17, 2023.

### 2.1 Eligibility criteria

All original research papers on barriers and facilitators to the implementation of One Health interventions and strategies in developing countries meeting the following eligibility criteria were included: (1) research with a quantitative, qualitative or mixed design; (2) articles published in English only; (3) articles published between 2008 and 2023, after the adoption of One Health in December 2007 by the tripartite alliance between FAO-OIE-WHO; (4) limited to low- and middle-income countries based on the list provided by the World Bank in June 2022 (21).

The following exclusion criteria were considered: (1) review articles, commentaries, letters, discussion papers, posters, conference abstracts, conference reports, dissertations and systematic reviews; (2) Any article whose full text was not available.

### 2.2 Sources of information

A complete search strategy was developed to identify studies published in English from January 2008 to January 2023. Five databases were used, namely PubMed, CINAHL, Global Health, Embase, and Web of Science.

### 2.3 Search strategy

The search strategy we used in the PubMed database is as follows in Table 1. This search equation has been adapted for the following electronic bibliographic databases: Embase, Global Health Web of Science and CIHNAL.

**Table 1:** PubMed search strategy.

((((((Environment[MeSH Terms]) OR (Humans[MeSH Terms])) OR (Animals[MeSH Terms])) OR (Ecosystem[MeSH Terms])) OR (Plants[MeSH Terms])) AND ((((((One Health[MeSH Terms]) OR (“Intersectoral Collaboration” [MeSH Terms])) OR (“multisectoral health”)) OR (“ecosystem health”)) OR (ecohealth)) OR (“Intersectoral health”))) AND ((((((“Health Plan Implementation” [MeSH Terms]) OR (challenge*)) OR (obstacle*)) OR (“helping factors”)) OR (barrier*)) OR (facility*)) AND (“Developing Countries”[MeSH Terms])

### 2.4 Registration of studies

We performed a first search, and the results were imported into Rayyan. Duplicate items were cleaned up first after import. Then, three groups of two reviewers (DSY and MMD) (GMK and RWS) and finally (OT and SF) independently made the first selection of titles and abstracts to exclude articles not relevant to our review based on our eligibility criteria. In the end, a consensus session was held to validate the selection. When discrepancies were not settled between the two reviewers, a third opinion was requested. Next, the full-text articles to be reviewed were imported into ZOTERO software, and a second screening was done by three groups of two reviewers each (DSY and MMD), (GMK and RWS), (OT and SF). A second working session was organized to validate and harmonize this selection, and a third opinion resolved the discordances. Finally, a flowchart was made to summarize the article’s selection process.

Following the first search, results were imported into Rayyan software and duplicate items were removed. Three pairs of reviewers (DSY and MMD) (GMK and RWS) and (OT and SF) independently screened titles and abstracts to exclude non-relevant articles based on the eligibility criteria. At the end of this step, a consensus session. When the discrepancy was not settled, an opinion of the senior research member (PN) was requested. The full texts of selected articles were imported into ZOTERO software, and the pairs of reviewers independently did a second screening. A second working session for a consensus on this step was held, and PN’s opinion was sought for the discordances. A flowchart was developed to summarize the study selection process.

### 2.5 Data elements

An extraction grid was developed by a reviewer (DSY) and validated by all team members. It was used to extract the following different variables: (1) DOI or PMID of the article; (2) Title of the study; (3) Initials of the reviewer; (4) First authors’ name; (5) Year of publication; (6) Countries involved; (7) Location where the study was conducted; (8) Objectives of the study (9) Field of intervention (10) Study design (12) Period of study (13) Collect data period (14) Sectors implied (15) Participants (16) Data collection method (17) Data collection tools (18) Variables (19) Analysis method (20) Population (21) Main findings related to barriers (22) Main findings related to enablers factors.

### 2.6 Results and prioritization

The main results are the barriers and facilitating factors for implementing One Health interventions. We have categorized these at four levels: national, regional, district, and community.

An extraction grid was developed and validated by all team members. The following data were extracted: (1) DOI or PMID of the article; (2) Title of the study; (3) Initial of the reviewer; (4) First author name; (5) Year of publication; (6) Countries involved; (7) Location where the study was conducted; (8) Objective of the study (9) Field of intervention (10) Study design (12) Period of study (13) Collect data period (14) Sectors implied (15) Participants (16) Data collection method (17) Data collection tools (18) Variables (19) Analysis method (20) Population (21) Main findings related to barriers (22) Main findings related to facilitators.

### 2.7 Risks of bias of individual studies

We assessed the quality of the articles with the 2018 version of the Mixed Methods Appraisal Tool (MMAT). The MMAT is a critical appraisal tool for qualitative, quantitative, and mixed-methods studies. It assesses five categories of methodological quality: qualitative research, randomized controlled trials, non-randomized studies, quantitative descriptive studies, and mixed methods studies (22–25).

### 2.8 Data synthesis

To synthesize our findings, we performed a thematic analysis. The analysis and synthesis used the public health emergency preparedness and response framework (26). We chose to use it because it responds to complex health problems such as One Health. This framework outlines 12 elements that play a role in the complex public health emergency management system. They are (01) governance and leadership, (02) planning process, (03) collaborative network, (04) community engagement, (05) risk analysis, (06) monitoring and monitoring, (07) practice and experience, (08) resources, (09) workforce capacity, (10) communication, (11) learning and evaluation, (12) ethics and values. We chose it because it responds to complex health problems such as One Health. The main results are the barriers and facilitating factors for implementing One Health interventions. We have categorized these at four levels: national, regional, district, and community.

## 3 Results

### 3.1 Included studies

**Figure 1:**
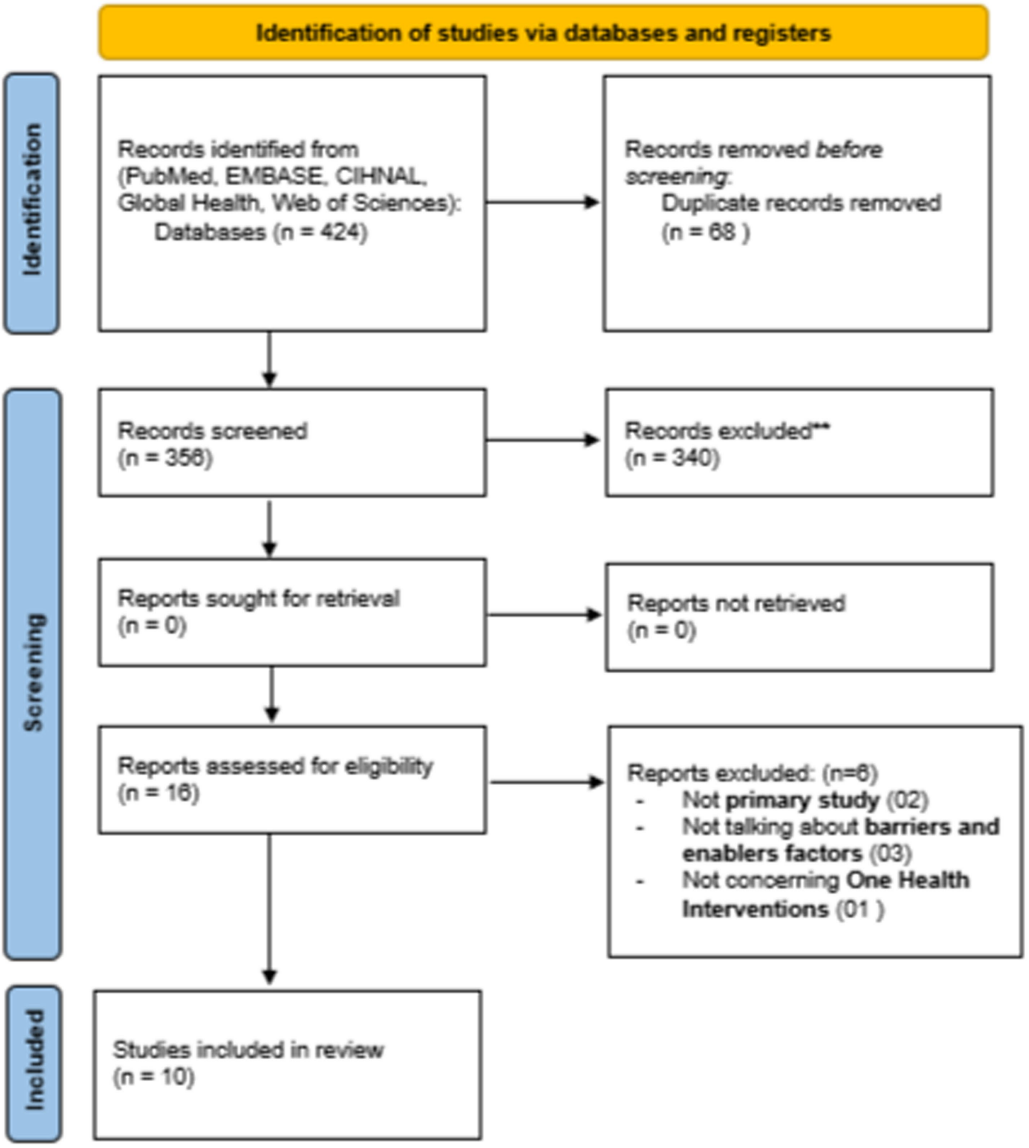
PRISMA flow diagram of the study selection (2020).

A total of 424 articles were initially identified through the electronic database search. After removing duplicates (*n* = 68), 356 articles were screened for title and abstract, and 16 were retained for full-text screening. Of these, six (*n* = 6) articles were excluded for the following reasons: not primary study (*n* = 2), not talking about barriers and enablers (*n* = 3), and not reporting a One Health intervention (*n* = 1). The study selection process following the PRISMA diagram is illustrated in Figure 1.

### 3.2 Study characteristics

The articles in our systematic review used different designs, including qualitative studies, qualitative descriptive analyses, mixed methods, literature reviews, cross-sectional surveys, and Rapid Rural Appraisal (RRA). The duration of the study and the periods of data collection varied. The most common study design utilized across all studies was a mixed-method study (*n* = 6). The included articles were published as early as 2008 and as recently as 2022. The ten studies were conducted in Tanzania (*n* = 4), Chad (*n* = 1), Uganda (*n* = 1), Kenya (*n* = 1), India (*n* = 1), Burkina Faso (*n* = 1) and the last one included Nigeria, Tanzania, and Uganda. They all had as an objective, among others, the determination of the facilitators and the real obstacles to the collaboration between several specialists for implementing a one health approach for better management and control in several fields of intervention, including the fight against zoonotic diseases and mass vaccinations.

### 3.3 Quality assessment of the articles included

For the assessment of the quality of the included articles, we used the MMAT Evaluation. The methodological quality of the 10 studies included in this synthesis was assessed using the Mixed Methods Appraisal Tool (MMAT). The MMAT is a critical appraisal tool designed for mixed systematic reviews, i.e., reviews that include quantitative, qualitative, and mixed methods studies. It allows the appraisal of five methodological quality categories: qualitative research, randomized controlled trials, non-randomized studies, quantitative descriptive studies, and mixed methods studies. The tool is divided into two parts. First, the tool was suited for this review as it was explicitly developed for quality appraisal in systematic reviews involving qualitative, quantitative and mixed methods designs. The MMAT criteria list includes indicators that explain and illustrate some criteria. Six of the 10 articles assessed were mixed, and two were qualitative. The studies involved experts and specialists from the human, animal and wildlife sectors and policymakers. For each question, the authors responded by checking “Yes” or “No,” “Cannot tell,” and “comments”. In terms of the methodological quality of the articles, a total of five studies scored 5/5 (100% Very high quality), two studies scored 4/5 (80% High quality), and three studies scored 3/5 (60% Medium quality). In general, the articles evaluated had excellent MMAT scores (n = 5), ranging from 60% (n = 1) to 100%, indicating an overall high methodological quality (see Table 2).

**Table 2:** Criteria from the mixed methods appraisal tool.

Title of the study	1. Qualitative					5. Mixed methods				
	1.1	1.2	1.3	1.4	1.5	1.1	1.2	1.3	1.4	1.5
1_ “Using the same hand”: The complex local perceptions of integrated one health-based intervention in East Africa						**1**	**1**	**1**	**?**	**1**
2_Evaluation of the feasibility and sustainability of the joint human and animal vaccination and its integration into the public health system in the Danamadji health district, Chad						**1**	**1**	**1**	**1**	**1**
3_ After-action review of rabies and anthrax outbreaks multisectoral response in Tanzania, challenges						**1**	**1**	**1**	**?**	**?**
4_ Implementing One Health Concept in rural communities: approaches and Challenges in Sierra Leone						**1**	**?**	**1**	**1**	**?**
5_ Practice of One Health Approaches: Bridges and Barriers in Tanzania	**1**	**1**	**1**	**1**	**1**					
6_ Experiences of the one-health approach by the Uganda Trypanosomiasis Control Council and its secretariat in the control of zoonotic sleeping sickness in Uganda						**1**	**?**	**1**	**1**	**?**
7_ Cross-Sectoral Zoonotic Disease Surveillance in Western Kenya: Identifying Drivers and Barriers Within a Resource-Constrained Setting	**1**	**1**	**1**	**1**	**1**					
8_ Operationalizing the “One Health” approach in India: facilitators of and barriers to effective cross-sector convergence for zoonoses prevention and control						**1**	**1**	**1**	**?**	**1**
9_ One Health: Past Successes and Future Challenges in Three African Contexts	**1**	**1**	**1**	**1**	**1**					
10_ Toward integrated surveillance of zoonotic diseases in Burkina Faso: the case of anthrax	**1**	**1**	**1**	**1**	**1**					

### 3.4 Different one health interventions done

Our results allowed us to identify different One Health strategies related to neglected tropical diseases, immunization, the nature and scope of collaborative arrangements between human, animal, and wildlife health experts in an intersectoral partnership, and the control of specific diseases. A wide range of empirical studies was examined, the most common being challenges and approaches to implementing the One Health initiative, general gateways for and barriers to collaboration from the perspectives of health experts, and finding actual gateways for and barriers to collaboration among the health experts interviewed, determining the proportions of health experts who have collaborated with other experts from disciplines different from their own. The different sectors that our study focused on were the human health sector, animal health sector, environment, political science, wildlife sector and social work sectors.

### 3.5 Barriers and enablers factors

Implementing One Health interventions/strategies in Africa is a major public health challenge for preventing and controlling emerging diseases. However, this implementation can be hampered by numerous barriers, the understanding of which is essential to developing effective policies and strategies. However, Africa also presents facilitators for implementing One Health interventions/strategies in Africa.

#### 3.5.1 Barriers

The thematic analysis results identified several barriers to implementing One Health interventions/strategies in Africa (see Table 3).

**Table 3:** Different barriers found.

Barriers summary	
*Governance and leadership* National (27–31) Regional (32) District (33) Community (34)	_Lack of political will, unsupportive policies _Lack of organized institutions and a proper and legal framework. _Lack of policies in different sectors _Duplication of other institutional arrangements _Bureaucracy and coordination challenges _Problem of ownership by ministries _Underbudget for the support of the function of the approach _Unproper decisions taken at the central level, not adapted to the realities in the field _The platform is not fully operational to meet the objectives _Lack of interest of health experts in the “one health” concept _Low level of adoption of One Health approaches was low (in the community), _No sops for preparedness and response available at the local level
*Planning process* District (34)	_No guidelines for preparedness and response _No up-to-date information on preparedness and response protocols and availability of emergency resources such as PPE vaccines, syringes etc. was often poor
*Collaborative networks* National (28, 29) District (33, 34) Community (27, 34, 35)	_Competing departmental priorities and institutional interests _Shortcomings in collaboration for intersectoral surveillance _No evidence of formal multisectoral collaboration and communication _Lack of teamwork between health experts _Egoism of health experts who do not consider public benefits when collaborating _Lack of data sharing with other sectors _Lack of trust in drugs and the intervention by the community _Lack of communication with the community _Weak implication of animal health sector response in the community
*Community engagement* At the local level (35)	_Hesitancy due to hygienic problems (mode of delivery)
*Surveillance and monitoring* National (28, 31) Regional (28) District (29, 34) Community (34)	_Disparate human and animal disease reporting systems, _Weak health system in the animal and human sector _Absence of an effective data-sharing system at all the level _Weak mainstreaming in the disease surveillance system _Lack of adequate resources, particularly for the detection of cases in event-based surveillance systems _Not enough staff to cover the whole territory effectively _High turnover of officers at the local level exacerbates the lack of skilled maintenance _Weakness of laboratory network _The paper-based alert system is considered too archaic to allow for rapid and quality notification and response
*Practice and experience* National (28, 29) District (29) Community (29, 35)	_Limited knowledge of zoonoses by relevant cross-sector actors _Lack of training and technical capacities and lack of appropriation of the concept by some sectors like environment and animal _Low level of implication in the environment and animal sector in surveillance and interventions _Lack of support of another sector, like the environment, by TFPs _Lack of dissemination of the approach within the various institutions in the central and decentralized services _Lack or low level of knowledge about how joint interventions at the local level _Communities are less well aware than the central level in One Health activities
*Resources* National (27–30, 34) Regional (29, 32) District (33, 34) Community (34)	_Insufficient financial resources from the government _Mismanagement or lack of funds and supplies for emergency and disease investigation _Poor infrastructure and resourcing, particularly in human and animal health service _Lack of human resources _Lack of cold chain maintenance of the vaccine _Lack of adequate resources for the function of One Health Platform activities _The TFPs more frequently tend to finance vertical programs for specific diseases, particularly in the human sector _Weakness of the national capacity to mobilize resources
*Workforce capacity* National (27, 29)	_Lack of trust toward local health systems _Mistrust between actors _Egos and different mindsets among actors _Different administrative cultures or working practices
*Communication* National (27–29) Regional (32) District (27) Community (27, 35)	_Lack of information and communication for the activities to put in place in the community or asymmetries of information _Lack of telephone coverage in certain areas, which hinders the proper circulation of information _Problem of leadership during investigation missions and the ability to work as a team remains difficult in the field __Absence of formal channels of communication __Low-risk awareness of zoonotic activities at all the level __Not enough communication awareness about drugs and One Health intervention with the community

##### 3.5.1.1 Governance and leadership

Our review showed that weak governance and leadership were barriers to implementing One Health interventions in Africa. As a result, local governments have not been able to effectively coordinate the various stakeholders’ actions and mobilize sufficient resources to support the implementation of such interventions.

##### 3.5.1.2 Planning process

Inadequate planning was also a significant barrier to implementing One Health interventions in Africa. Plans need to be more detailed to provide clear guidance to stakeholders involved in implementation, making it difficult to set clear and measurable objectives and measure progress.

##### 3.5.1.3 Collaborative networks

Ineffective collaborations between stakeholders were identified as a major barrier to implementing One Health interventions in Africa. The various stakeholders involved in implementing these interventions often work in isolation, without a comprehensive collaboration system, which can lead to unnecessary duplication of effort and resources and ineffective actions.

##### 3.5.1.4 Community engagement

More engagement of local communities has also been identified as a significant barrier to implementing One Health interventions in Africa. This is because local communities often need to be sufficiently involved in the planning and implementation, leading to poor buy-in and stakeholder communication.

##### 3.5.1.5 Surveillance and monitoring

Inadequate disease surveillance and monitoring are significant barriers to implementing One Health interventions in Africa. Surveillance systems often need to be better developed or more present, making detecting diseases early and responding quickly tricky.

##### 3.5.1.6 Practice and experience

Lack of practice and experience was a significant barrier to implementing One Health interventions in Africa. Stakeholders involved in implementation often need more training and practical experience, which can lead to mistakes and inadequate implementation.

##### 3.5.1.7 Resources

The need for more resources was a significant barrier to implementing One Health interventions in Africa. However, the financial and human resources needed to implement these interventions effectively are often insufficient or poorly allocated, which can lead to delays or interruptions in implementation.

##### 3.5.1.8 Workforce capacity

Lack of specific skills, inadequate training, limited staffing, and unattractive salaries in the animal and public health fields are challenges to implementing One Health interventions. In addition, frequent turnover of public health workers and migration of health professionals to high-income countries also hinder the development of qualified and experienced staff for One Health interventions.

##### 3.5.1.9 Communication

The results indicate that communication is often ineffective and limited between the actors involved in One Health interventions in Africa, particularly between human and animal health professionals. This can lead to difficulties in developing joint action plans, coordinating disease surveillance and control activities, and raising community awareness of the importance of human, animal and environmental health. The studies also highlight the need to strengthen communication and collaboration among different stakeholders to support the successful implementation of One Health interventions in Africa.

These findings suggest that implementing One Health interventions/strategies in Africa faces several complex and interconnected barriers that require an integrated and coordinated approach.

#### 3.5.2 Enablers factors

The results also highlighted several facilitating factors (see Table 4) for the implementation of One Health interventions/strategies in Africa.

**Table 4:** Different enablers factors found.

Facilitators summary	
*Governance and leadership* National (27, 28, 31, 34, 36) Regional (32) District (33)	_Existence of a reference framework document _Existence of framework for reflection, orientation, and planning of health activities _Political good willingness of One Health intervention _Existence of the national One Health platform well-coordinated with multi-sectoral emergency response _Existence of virtual and physical meetings conducted by a multisectoral platform _Organization of joint and field simulation exercises to test the capacity for preparedness and response frameworks _Coordination group for the response with all sectors _Existence government leadership _Formal governance and leadership structures _High-level political backing _Strong political commitment _Existence of multisectoral and common objectives _Strong political will
*Collaborative networks* National (27–30) Regional (34) District (33, 34)	_ Good coordination between the public health and veterinary services at the central and decentralized level _Clear delineation of sectoral roles _Developing clear operational guidelines and frameworks for cross-sectoral collaboration _Energized communication between the Ministries of Health and Agriculture, awareness, and mutual professional respect _ Motivation of stakeholders to engage in collaboration for the surveillance _The culture of collaboration and collective interest _The existence of a shared vision _Trust based on respect and recognition _ Good governance of intersectoral surveillance _Existence of joint meeting that advocated ONE HEALTH approach with the implication of human, animal environmental sectors, and other institutions _Existence of district health emergency response teams _Coordination of the resource’s mobilization
*Community engagement* National (27, 31) Community level (27, 35)	_Implication of community _Consideration of food, environmental, social needs, religious, and cultural aspects before the intervention _ Orientation and education of people on good animal husbandry practices, the role and importance of the dog in providing security, and in the control of dog-associated rabies _Ensure good working relationships between animal and crop farmers in the context of One Health _Implication of anthropologist _Identify the need of the target population _Involvement in social mobilization _The benefit gain: cost saving, health benefits, gain of time, many services at the same time, and efforts _Knowledge and awareness of the importance of One Health intervention for the community
*Surveillance and monitoring* National (29, 31, 36) Regional (32) Community (34)	_Identification of the diseases of interest for the implementation _Presence of an effective data sharing system at all the level _Knowledge of the epidemiological cycles of diseases, which demonstrate the link between human, animal, and environmental compartments _Transfer of sample from health facility to zonal veterinary laboratory
*Practices and experience (learning)* National level (29) Regional (34) District (33, 34)	_Good knowledge of surveillance actors about the One Health concept _Good awareness of the importance of collaboration and multi-partner and ONE HEALTH approach to managing complex health problems _Retooling courses concerning the OH approach
*Resources* National (27, 28, 30, 31) Regional (32, 34) District (33) Community (27)	_ Funder’s adherence to the approach and existence of financial support from donors _Availability of personnel, resources, and fund _Trained and enough personnel _ Good resourcing considerations _ Ongoing financial support to weather the inter-ministerial “turf wars.” _ Mobilization of national budgets _Availability of qualified vaccinators and supervisors (actors) at the local level
*Communication* National (28, 36) Regional (32, 33) District (33, 34)	_Communication, information sharing _Building a joint communication platform for data sharing on zoonoses based on existing human and animal disease reporting systems _Leverage on past and ongoing collaborative mechanisms _Existence of good formal and information communication _Presence of formal channels of communication _Efforts of communication between human and animal sectors when diseases occurred _Good information exchange _Awareness of the “One Health” concept among policymakers

##### 3.5.2.1 Governance and leadership

Several studies have highlighted the importance of strong governance and effective leadership to facilitate the implementation of One Health interventions/strategies in Africa. Multi-sector partnerships and inter-institutional coordination are crucial elements of effective governance.

##### 3.5.2.2 Planning process

Planning was critical for successfully implementing One Health interventions/strategies in Africa. The studies highlighted the need for strategic planning, operational plans, and participatory planning to ensure effective implementation.

##### 3.5.2.3 Collaborative networks

Collaborative networks were identified as a critical factor for successfully implementing One Health interventions/strategies in Africa. Public-private partnerships, the collaboration between the human and animal health sectors, and collaboration between communities were critical elements of successful collaboration.

##### 3.5.2.4 Community engagement

Community engagement was critical for successfully implementing One Health interventions/strategies in Africa. The studies emphasized the importance of involving communities in designing and implementing One Health interventions/strategies to ensure greater acceptance and buy-in.

##### 3.5.2.5 Surveillance and monitoring

Surveillance and data collection are vital in effectively ensuring the implementation of One Health interventions/strategies in Africa. The studies highlighted the importance of integrated surveillance and cross-sectoral data sharing for timely and appropriate decision-making.

##### 3.5.2.6 Practice and experience

Practice and experience were crucial factors for successfully implementing One Health interventions/strategies in Africa. The studies highlighted the importance of training, capacity building, and practical experience for human and animal health professionals to ensure effective implementation.

##### 3.5.2.7 Resources

Financial and material resources were critical factors for successfully implementing One Health interventions/strategies in Africa. The studies emphasized the importance of adequate funding, material resources, and access to technology to ensure effective implementation.

##### 3.5.2.8 Workforce capacity

Building the capacity of human and animal health personnel was identified as a critical factor for the successful implementation of One Health interventions/strategies in Africa. The studies emphasized the importance of well-trained, well-equipped, and motivated staff to ensure effective implementation.

##### 3.5.2.9 Communication

Effective communication was critical for successfully implementing One Health interventions/strategies in Africa. The studies emphasized the importance of open and transparent communication between the different actors and effective communication with communities to ensure successful implementation.

## 4 Discussion

### 4.1 Summary of the main findings

The concept of One Health has become increasingly important over the past decade as global health challenges continue to escalate. One Health recognizes the interconnectedness of people, animals, and the environment and seeks to promote collaboration across sectors to optimize health outcomes (6). However, despite the potential benefits of One Health interventions, barriers often hinder their implementation. This review systematically identified barriers and enablers factors to implementing One Health interventions or strategies. We use the resilience framework for public health emergencies to address them adequately. We noticed that several elements were mentioned.

The first barrier to implementing One Health interventions in developing countries is the need for more political will. Some documents discussed weak governance, the absence of a regulatory environment, and the need for more consensus on priority setting. We also notice a need for more guidelines for preparedness and response during the planning process as a great problem. There needed to be formal collaboration and communication between sectors. Concerning the surveillance aspect, the weakness of the surveillance system was indexed with limited knowledge of the One Health approach by sectors and the community. One of the most significant barriers was the need for more human, material or logistical resources. For some papers, limited financial resources or inadequate use of funding was critical for the successful implementation of One Health interventions. The approach also requires a collaborative effort from different sectors, which could have diverse funding sources and pose a challenge. Mistrust and limited cooperation among stakeholders, including the community, are also huge barriers. Concerning the enablers factors, the existence of reference framework documents and programs for reflection, orientation and planning of One Health activities had a positive impact. Good coordination between the different sectors at the different levels was also found. Joint and multisectoral meetings that advocated the One Health approach were considered crucial. The existence of a good and formal platform of communication between the sectors was good. The Availability of funds and adequate resources coupled with the support of Technical and Financial partners that adhere to the approach was necessary.

### 4.2 Comparison with other reviews

This is the first study to synthesize barriers and enablers to implementing One Health interventions or strategies in developing countries. Barriers and facilitators have been systematically reviewed in other settings, but until now, syntheses have yet to be developed for the developing countries’ context. In addition, we structured the different barriers and facilitators according to the resilience framework for public health emergencies.

#### 4.2.1 Barriers

Weak national One Health coordination and few policies that support One Health interventions are challenges for exemplary implementation. Also, more political will and policy support is needed to solve this (37, 38). For example, a review of the One Health approach in Ethiopia showed that the leadership’s weakness and the higher government’s commitment are a great challenge (39). Also listed was that there needs to be more explicit legislation on the engagement of public-private partnerships with One Health (39). Also, most of the time, there are no existing public health frameworks to guide the operationalization of One Health in some low-resource countries, like some in Africa (40).

Many countries may be reluctant to invest in One Health initiatives as they may need an immediate return on investment. Additionally, political instability in some countries can make developing and implementing long-term strategies difficult.

A limited or inexistence collaboration across sectors can also affect One Health interventions and be a significant barrier. It corroborates the findings of the review of One Health challenges in Tanzania, in which the authors pointed out a gap, the integration of some sectors in the activities of another, rather than combined efforts (41). Another study explained that there is a feeble contribution and poor integration of some sectors, especially veterinary and environmental sectors, in collaboration, data sharing, and coordination of strategies (39, 40, 42). All these are emphasized because different sectors or government ministries may have different mandates, priorities, and policies, which can create tensions and hinder collaboration (43). These tensions may be exacerbated in developing countries with limited resources and capacity to facilitate collaborative efforts. In the case of Rwanda, solving One Health problem involving one sector at the expense of another has. As a result, the perception of ‘Winners” and “losers” (44).

Mistrust and limited cooperation among the partners could pose a challenge. According to the experience in India, neglecting the need to endorse linkages between human health, animal health and farming, agriculture, and environmental sectors has led to duplicative and weak response systems (45).

Capacity building is not done equally in the different sectors. Improvement of qualified individuals at different levels is important but insufficient (46). In some developing countries, capacities in human health are more built than in the veterinary sector, primarily because of competing priorities (45). In addition, some developing countries may need more education and awareness around One Health concepts. Implementing One Health interventions can make it challenging, as stakeholders may need help understanding the benefits or the importance of collaboration across sectors. A study by Ayobami et al. concerning implementing the One Health approach in Nigeria and other sub-Saharan African countries also showed that the sense and scope of One Health could be more precise to many stakeholders, which is a limitation to its implementation. In addition, at the technical level, the limited diagnostic capacity among researchers and public health laboratories is also a challenge (40).

One of the most significant barriers to implementing One Health interventions in developing countries is limited resources. Many low- and middle-income countries need help to provide essential human health services and address the health of animals and the environment because of funding constraints (40). This can make it challenging to prioritize One Health interventions, which may be seen as a lower priority than urgent public health issues. During a congress to highlight One Health challenges and opportunities in developing countries, it has been shown that four areas for capacity-building needs are affected such as risk management policies that respect transboundary and international guidelines, sustained capacity building of applicably and appropriately knowledgeable and skilled One Health personnel, accredited environmental and clinical diagnostic laboratories with an integrated and shared database, and improved use of existing natural resources and implementation plans based on cost–benefit analyses (47).

#### 4.2.2 Facilitators

Strong governance and leadership at the higher level are paramount for the easy implementation of One Health interventions. The success of One Health approach interventions is based on the policies that allow a multisectoral approach to issues of interest for all sectors. Governments must provide the necessary policy support to implement One Health interventions successfully. Policies should focus on improving human, animal, and environmental health while fostering cooperation and collaboration among different sectors. Formulating strategic documents and operationalizing the One Health secretariat is relevant to strengthen coordination (38). The existence of reference framework documents and programs for reflection, orientation, and planning of One Health activities had a positive impact. The identification of engaging technical working groups inside an elevated platform can boost the implementation of One Health Strategies (38, 39). The experience of India with emerging diseases such as avian influenza and Ebola virus led to the establishment of institutionalized collaborative frameworks in India to adopt a One Health approach to disease prevention and control (45).

Good coordination and collaboration between all the sectors and at the levels were pointed out as important elements for implementing good One Health strategies. Our review revealed the importance of joint and multisectoral meetings to address common One Health issues. This is in line with a review that shows that to raise awareness, it is essential to facilitate communication and interdisciplinary collaboration and organize joint meetings to solve significant issues (41). A workshop in Burkina Faso with experts showed that a good and formal communication platform between the sectors is crucial. Identifying One Health focal point from each sector or ministry is also advisable (48). The Availability of funds, adequate resources, and the support of Technical and Financial partners that adhere to the approach was necessary. A review of the progress of implementing a regional One Health Coordination Mechanism (R-OHCM) in West Africa showed that political commitment at regional meetings and the country’s adoption of regional frameworks were key strengths to its success of it (38).

Strong partnerships and collaborations among various stakeholders are essential. It testifies the effective stakeholder engagement, a key facilitator in implementing One Health interventions in developing countries. Engaging stakeholders from different sectors can increase collaboration, buy-in, and more effective interventions. Strong partnerships and collaborations make identifying the challenges easier and designing appropriate interventions such as outbreak investigations and response (42, 43). A comment made in the case of Ethiopia confirms that. Murphy et al. stated that Ethiopia’s success using the One Health approach depends on all Member Ministries’ commitment to supporting the National One Health Steering Committee (NOHSC) (49). Partnerships also enhance the sharing of resources, knowledge, and expertise, which help to address challenges. The experience of Rwanda revealed that international partnership is an excellent benefit for a country, even more when it involves all the sectors such as medicine, public health, veterinary medicine, agriculture and the environment (44).

In developing countries, capacity building and training are essential to building the skills and knowledge necessary to implement One Health interventions effectively. This can include training health workers in zoonotic disease diagnosis and management, environmental monitoring and risk assessment. In addition, technical assistance and mentoring programs can also be effective capacity-building strategies. The paper of Mbugi et al. confirms that a better understanding of the epidemiology of infectious diseases in human and animal sectors is needed to implement the One Health approach successfully. This will mainly be possible through education and training among health personnel (41).

The reinforcement of surveillance skills and the capacitation of diagnostic laboratories are significant. An adequate surveillance system with a strong laboratory network, whether at the national, provincial or subnational level, is paramount (47). During the One Health Zoonotic disease prioritization for multisectoral engagement in Burkina Faso, a principal recommendation made at the end of the workshop was to strengthen the capacity of the laboratory of human and animal sectors to diagnose the prioritized diseases and also reinforce the collaboration between the three sectors involved in One Health to ensure diagnostic testing. The awareness of One Health and leaders who work across disciplines and sectors rapidly institutionalize the One Health approach (39, 50).

Education and awareness of people so that they will change their behaviors and practices to achieve the intended goals are paramount. Education will help to create awareness among the public about the benefits of One Health interventions and how to implement them. It will also assist in reducing mistrust and resistance among the communities. Education and awareness will also help to enhance community engagement in the process. Engaging local communities and investing to raise the knowledge of the community about One Health can also lead to an increased understanding of One Health concepts and their benefits. A study done in Africa with pastoralists confirmed that. It has shown that the community living with their animals is more open to the approach because of the added value of the cooperation between human and animal health services (51). Another example is Rwanda, where the country has elaborated a network of community health workers to reinforce the One Health movement (44).

Financial support can be a critical facilitator in implementing One Health interventions in developing countries. Many low- and middle-income countries may need more resources to invest in One Health initiatives. According to a review made in Tanzania, the One Health approach provides the opportunity for joint global health initiatives to use resources optimally compared to use in one sector only (41). Financial support from international organizations or donor agencies can help address this gap and provide the resources necessary to implement One Health interventions effectively. Adequate financing is essential for the successful implementation of One Health interventions. Therefore, securing adequate funding sources is critical. Financial resources should be allocated adequately to all sectors interested in One Health interventions. Technical and financial partners’ support will help address the lack of funding (38).

### 4.3 Implications for the field

One Health intervention can significantly promote the health of people, animals, and the environment. However, there are often barriers that hinder their implementation in developing countries. By addressing these barriers and leveraging these facilitators, all the sectors at different levels can work toward achieving optimal health outcomes in all One Health issues. At the different levels of interventions, the most frequent barriers and facilitators identified in this review help to ameliorate the conceptualization for One Health approach strategies.

### 4.4 Limitations of the review

A strength of our review is that we synthesized information across different interventions that can be implemented in the One Health context. Nonetheless, our study has some limitations. First, we must assume the strength of only some of our studies as results. Secondly, we are still determining if we can access all the relevant studies because primarily low- and middle-income countries have unpublished and grey literature inaccessible to us. Lastly, we have omitted some valuable studies by including only articles published in English and French.

## 5 Conclusion

One Health strategies and interventions must be implemented widely to address the rising burden of emerging infectious diseases, zoonotic diseases, and antimicrobial resistance. One Health is a concept that recognizes the interconnectedness of human, animal, and environmental health and the need for multidisciplinary collaboration to address complex public health challenges. His strategies’ implementation faces both barriers and facilitators. While challenges such as the lack of awareness, funding, and governance remain prevalent, opportunities such as partnership and increasing public awareness provide a path to facilitate the widespread implementation of One Health strategies and interventions. The multidisciplinary nature of the One Health approach is essential to creating a sustainable, healthier, and safer environment for all. One of the most significant barriers is the lack of awareness and understanding of the One Health concept among stakeholders, which hinders the collaborative efforts required to address complex public health issues. Funding constraints, conflicting priorities, and institutional silos are other barriers that hinder the integration of One Health approaches. However, there are also considerable facilitators, such as increasing public awareness of the approach, raising good communication and a strong partnership between the various stakeholders, including public health professionals, veterinarians, environmentalists, policymakers, and the public. It is necessary to provide the infrastructure and funding to promote multidisciplinary research and education and encourage international collaboration to address global health challenges.

## Data availability statement

The datasets presented in this article are not readily available because: We used data from published primary studies. Datasets are available from the authors of the primary articles. Requests to access the datasets should be directed to do this, contact the corresponding authors listed in the primary articles.

## Author contributions

PN conceived and supervised the study. DY, DM, and GK drafted the manuscript and synthesized the results. DY, DM, GK, RWS, and SF developed the search strategy. All the authors selected the studies and extracted the data. Finally, DY, DM, GK, and PN extensively reviewed the manuscript. All the authors read, provided feedback, and approved the final version of the manuscript.
